# Supplementary material for: Ammonium is the preferred source of nitrogen for planktonic foraminifer and their dinoflagellate symbionts
Source: Proc Biol Sci. 2020 Jun 17;287(1929):20200620. doi: 10.1098/rspb.2020.0620 (PMC7329048; doi:10.1098/rspb.2020.0620)
Supplement: Figure S1 [file rspb20200620supp1.pdf]

Ammonium is the preferred source of nitrogen for planktonic foraminifer and their dinoflagellate symbionts

Charlotte LeKieffre, Howard J. Spero, Jennifer S. Fehrenbacher, Ann D. Russell, Haojia Ren, Emmanuelle Geslin, Anders Meibom

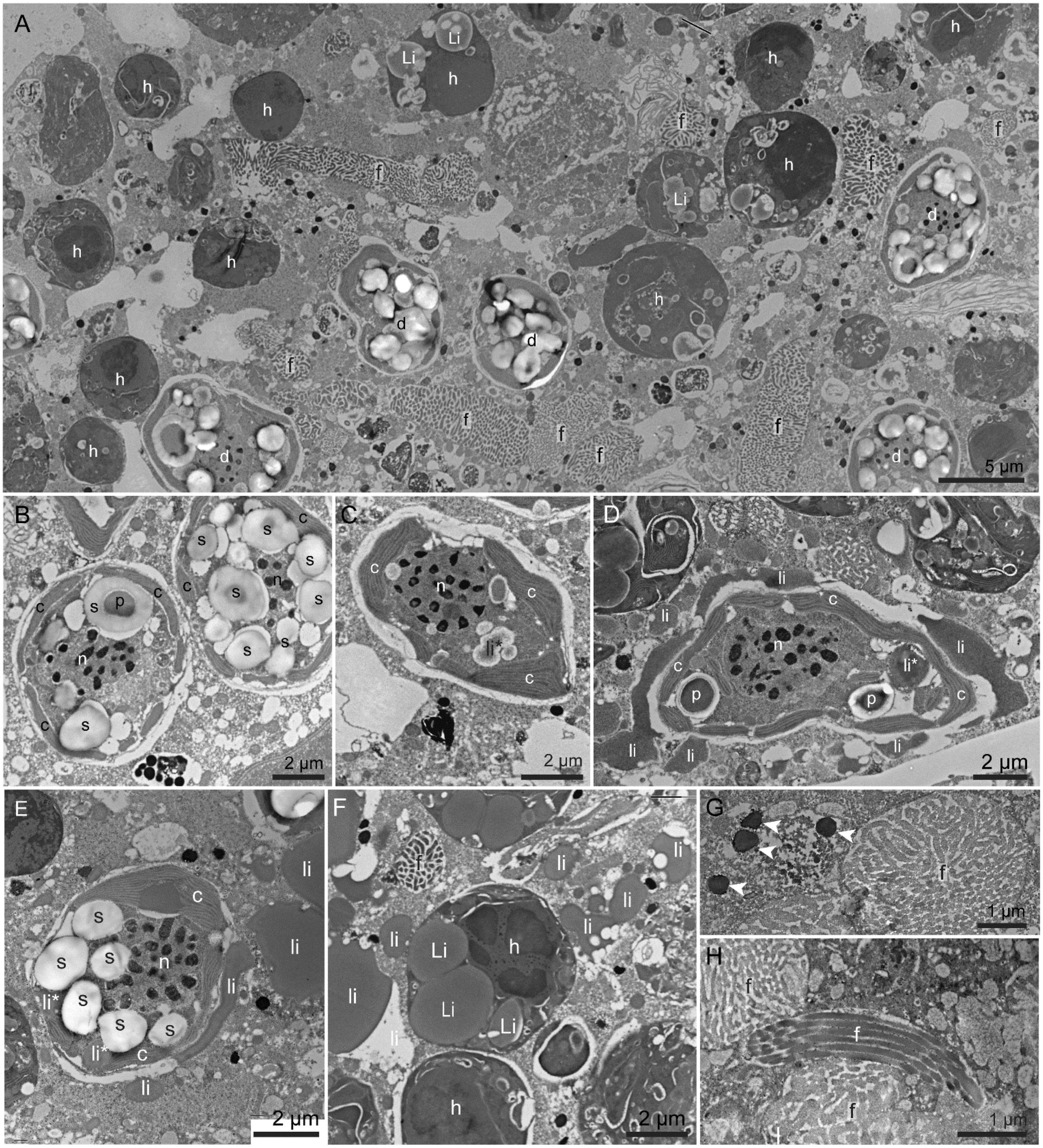

Figure S1: TEM micrographs of the cytoplasm and vacuolarized symbiotic dinoflagellates of *Orbulina universa*. A: Expanded view of the cytoplasm in a specimen sampled during the dark phase showing symbiotic dinoflagellates (d) within the vacuoles in the foraminiferal endoplasm, large electron-opaque heterogeneous vesicles (h), fibrillar bodies (f) and lipid droplets (Li). B: Symbiotic dinoflagellates observed in the foraminiferal endoplasm at the end of the light phase with starch (s) surrounding the chloroplast pyrenoid (p) in the left most symbiont cell and accumulated starch granules in the cell cytosol of the right most cell at t = 6 h. C: Symbiont at the end of the dark phase (t = 18 h). Note that all starch granules have been metabolized and are absent. D and E: Symbiotic dinoflagellates with lipid droplets in the cytoplasm and along their periphery or in the region between the dinoflagellate and the symbiosome (D: t = 1 h, E: t = 12 h). F: Lipid droplets and large heterogeneous vesicles in the foraminiferal endoplasm (t = 1 h). G: Higher magnification image of electron-opaque bodies (arrows) and fibrillar bodies. H: Higher magnification image of fibrillar bodies. Arrowheads: electron-opaque bodies, c: dinoflagellate chloroplast, d: dinoflagellate, f: fibrillar body, h: heterogeneous large vesicle, li: lipid droplet free in the endoplasm, Li: lipid droplet included in a large heterogeneous vesicle, li*: dinoflagellate lipid droplet, n: dinoflagellate nucleus, p: dinoflagellate pyrenoid, s: dinoflagellate starch grain.
