# Supplementary material for: Ammonium is the preferred source of nitrogen for planktonic foraminifer and their dinoflagellate symbionts
Source: Proc Biol Sci. 2020 Jun 17;287(1929):20200620. doi: 10.1098/rspb.2020.0620 (PMC7329048; doi:10.1098/rspb.2020.0620)
Supplement: Figure S2 [file rspb20200620supp2.pdf]

Ammonium is the preferred source of nitrogen for planktonic foraminifer and their dinoflagellate symbionts

Charlotte LeKieffre, Howard J. Spero, Jennifer S. Fehrenbacher, Ann D. Russell, Haojia Ren, Emmanuelle Geslin, Anders Meibom

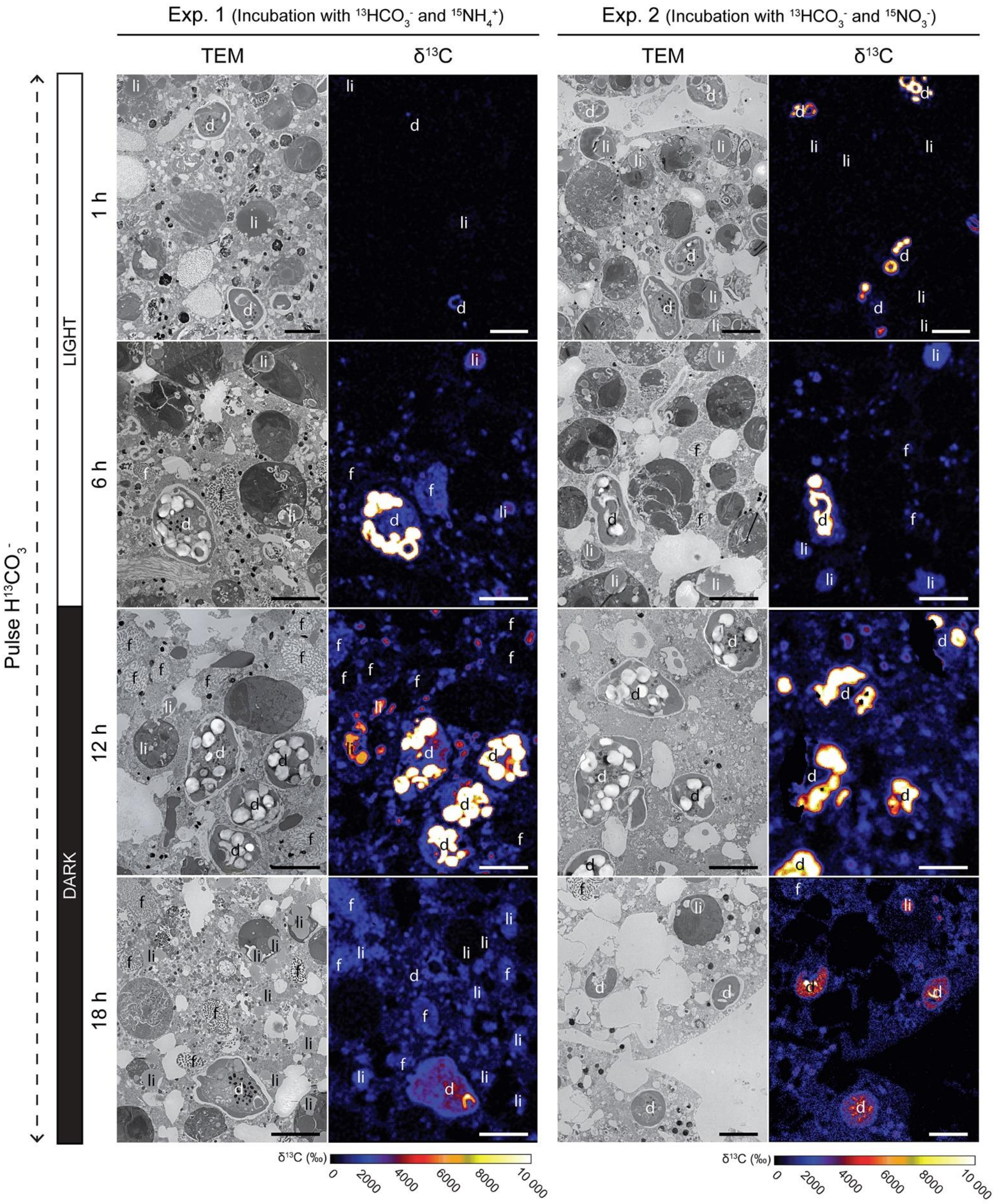

Figure S2: Time-evolution of ^{13}C assimilation and starch production/accumulation in the *Orbulina universa* symbiotic dinoflagellate endoplasm during the light (t = 1 and 6 h) and dark phases (t = 12 and 18 h). TEM micrographs of *O. universa* endoplasm with symbiotic dinoflagellates and corresponding NanoSIMS images of $^{13}C/^{12}C$ distributions (expressed as $\delta^{13}C$ in ‰). Left columns: incubation in $H^{13}CO_3^-$ and $^{15}NH_4^+$ Exp. 1. Right columns: incubation in $H^{13}CO_3^-$ and $^{15}NO_3^-$ Exp. 2. d: dinoflagellate, f: fibrillar body, li: lipid droplet. Objects appearing white in NanoSIMS images are starch deposits with ^{13}C-enrichments above the imposed 10 000 ‰ upper color scale limit. Scale bars: 5 µm.
