# Supplementary material for: Ammonium is the preferred source of nitrogen for planktonic foraminifer and their dinoflagellate symbionts
Source: Proc Biol Sci. 2020 Jun 17;287(1929):20200620. doi: 10.1098/rspb.2020.0620 (PMC7329048; doi:10.1098/rspb.2020.0620)
Supplement: Figure S3 [file rspb20200620supp3.pdf]

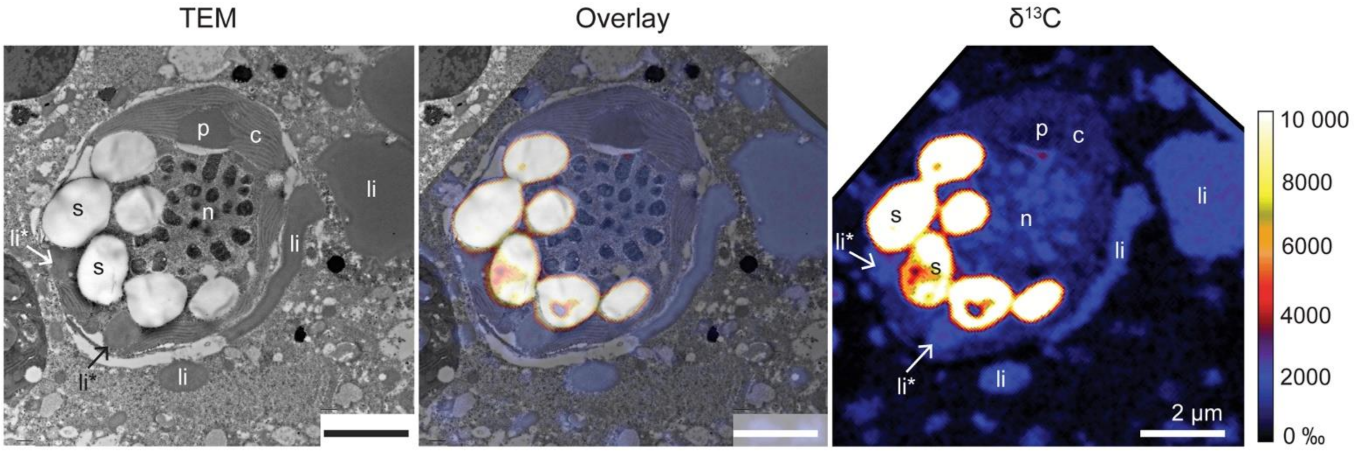

Figure S3: Lipid droplet translocation between a dinoflagellate and the foraminiferal host cell. TEM micrograph and corresponding $\delta^{13}C$ NanoSIMS images of the symbiotic dinoflagellate presented in panel E of Figure S1 (t = 12 h, $\delta^{13}C$ expressed as ‰). Objects appearing white in NanoSIMS images are starch deposits with ^{13}C-enrichments > 20 000 ‰ (upper color scale limit). d: dinoflagellate, li: lipid droplet free in the endoplasm, li*: dinoflagellate lipid droplet, n: dinoflagellate nucleus, p: dinoflagellate pyrenoid, s: dinoflagellate starch grain.
