# Supplementary material for: Ammonium is the preferred source of nitrogen for planktonic foraminifer and their dinoflagellate symbionts
Source: Proc Biol Sci. 2020 Jun 17;287(1929):20200620. doi: 10.1098/rspb.2020.0620 (PMC7329048; doi:10.1098/rspb.2020.0620)
Supplement: Figure S4 [file rspb20200620supp4.pdf]

Ammonium is the preferred source of nitrogen for planktonic foraminifer and their dinoflagellate symbionts

Charlotte LeKieffre, Howard J. Spero, Jennifer S. Fehrenbacher, Ann D. Russell, Haojia Ren, Emmanuelle Geslin, Anders Meibom

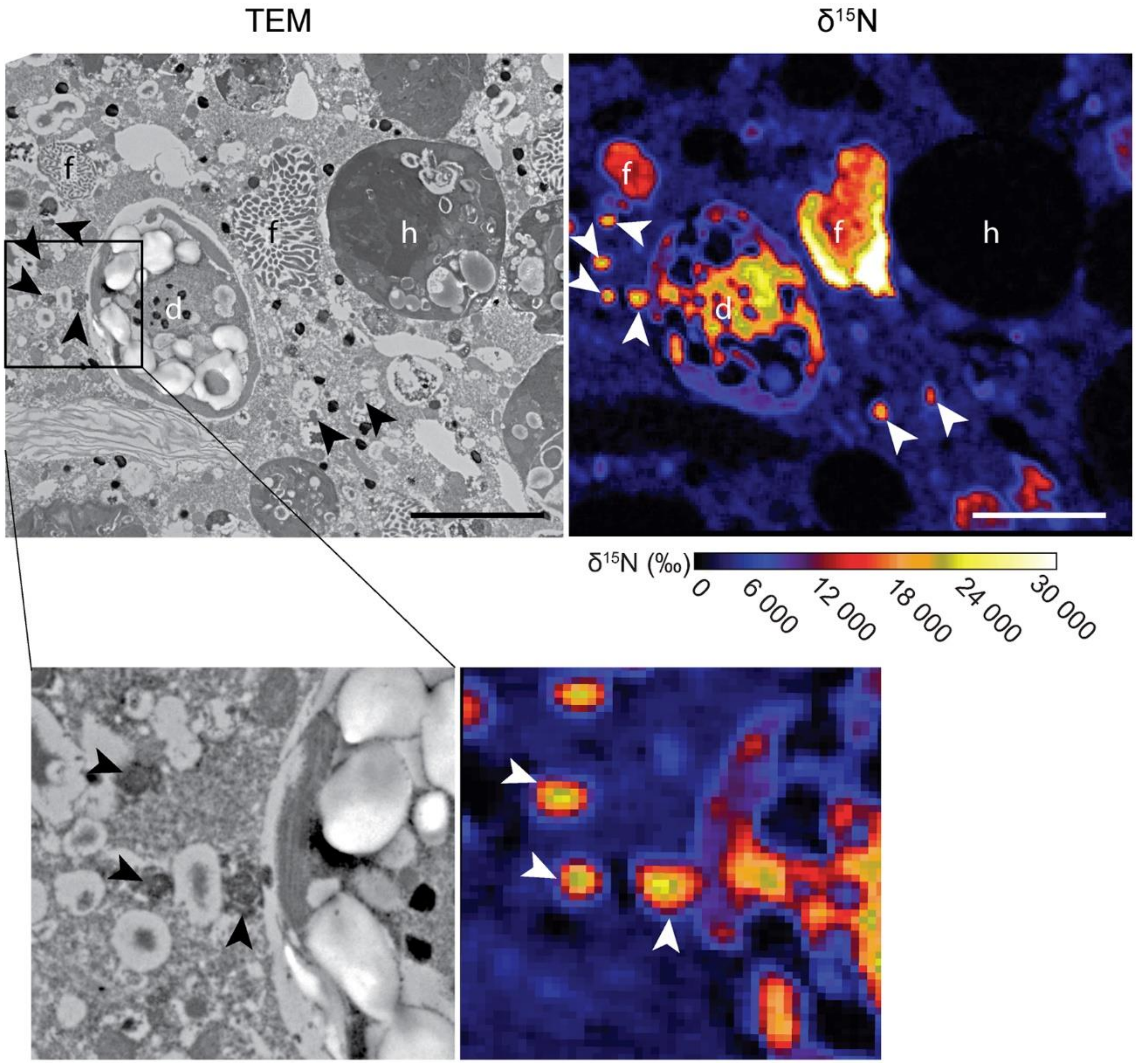

Figure S4: Highly enriched ^{15}N-labeled vesicles in the foraminifer endoplasm at the periphery of the dinoflagellate. High magnification image of boxed region in Figure 2 that shows regions of elevated ^{15}N accumulation at the end of the light phase (t = 6 h). The ^{15}N enriched regions correspond to sections of the dinoflagellate nucleus and chloroplast, and an *Orbulina universa* fibrillar body where extensive protein and nucleotide synthesis occurred during the light phase. Arrowheads: ^{15}N-enriched vesicles, d: dinoflagellate, f: fibrillar body, h: large heterogeneous vesicle. Scale bars: 5 µm.
