# Supplementary material for: Ammonium is the preferred source of nitrogen for planktonic foraminifer and their dinoflagellate symbionts
Source: Proc Biol Sci. 2020 Jun 17;287(1929):20200620. doi: 10.1098/rspb.2020.0620 (PMC7329048; doi:10.1098/rspb.2020.0620)
Supplement: Figure S5 [file rspb20200620supp5.pdf]

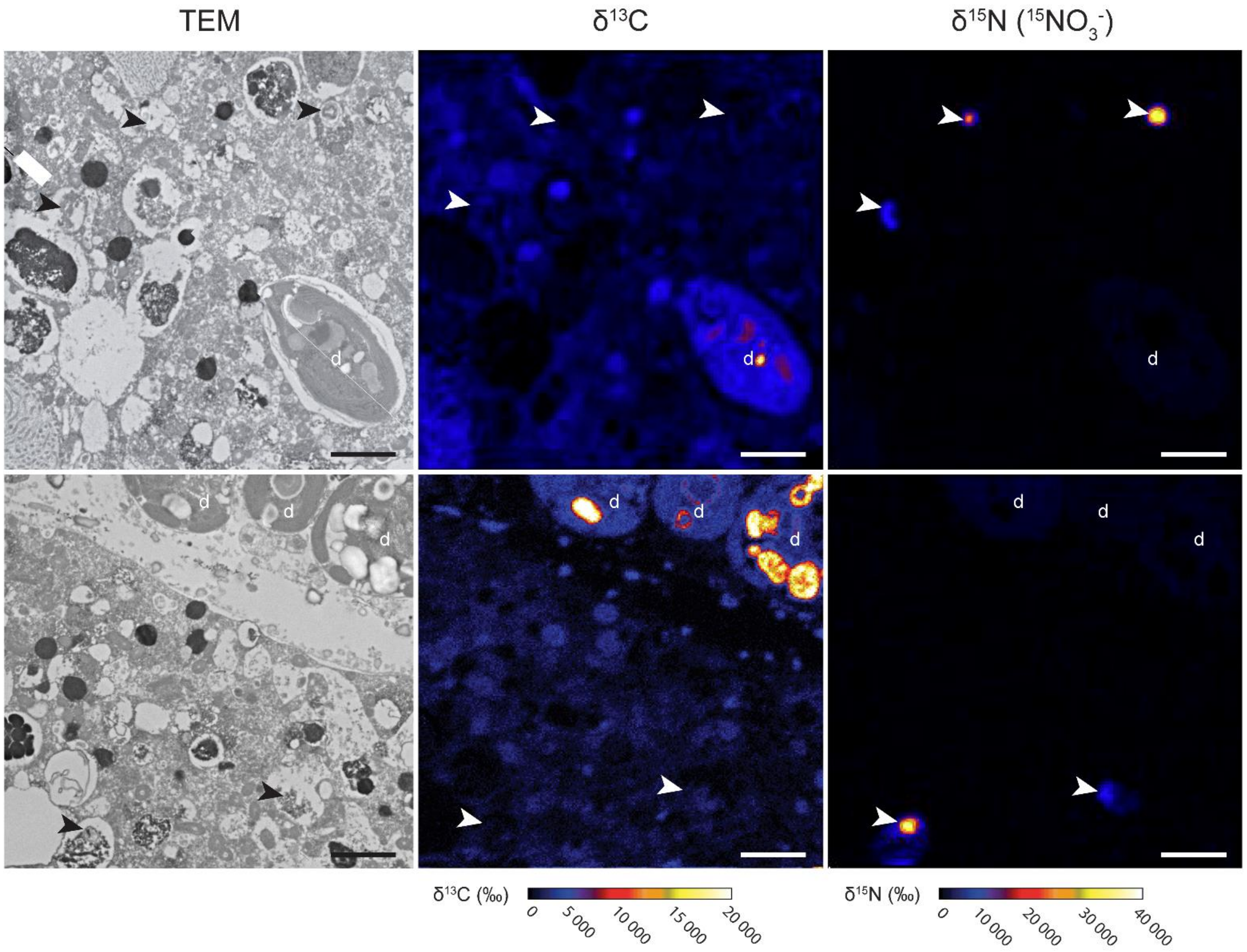

Figure S5: ^{15}N-enriched prokaryote-like vesicles in *O. universa* endoplasm. ^{15}N-enriched vesicles (potential prokaryotes in degradation vacuoles) in *O. universa* cytoplasm at t = 18h during Experiment 2 (incubation with $^{15}NO_3^-$). TEM micrographs are of *O. universa* cytoplasm and corresponding NanoSIMS images of $^{12}C/^{13}C$ and $^{15}N/^{14}N$ distributions (expressed as $\delta^{13}C$ and $\delta^{15}N$ in ‰). Arrowheads: highly ^{15}N-labeled vesicles, d: dinoflagellate. Scale bars: 2 µm.
