# Supplementary material for: Ammonium is the preferred source of nitrogen for planktonic foraminifer and their dinoflagellate symbionts
Source: Proc Biol Sci. 2020 Jun 17;287(1929):20200620. doi: 10.1098/rspb.2020.0620 (PMC7329048; doi:10.1098/rspb.2020.0620)
Supplement: Table S1 [file rspb20200620supp6.pdf]

Ammonium is the preferred source of nitrogen for planktonic foraminifer and their dinoflagellate symbionts

Charlotte LeKieffre, Howard J. Spero, Jennifer S. Fehrenbacher, Ann D. Russell, Haojia Ren, Emmanuelle Geslin, Anders Meibom

**Table S1**: List and characteristics of the specimens selected for experiment #1 (incubation with $^{15}NH_4$), experiment #2 (incubation with $^{15}NO_3$), experiment #3 (feeding with $^{13}C$-$^{15}N$-*Artemia*) and as controls for NanoSIMS analysis. Juvenile (j) refers to specimens fixed for TEM with only a trochospiral test, adult (a) refers to specimens fixed for TEM that had produced a spherical final chamber.

| Foram # | Experiment | Incubation time | Food particle when sampled | Life stage (juvenile / adult) | | Remarks |
|---|---|---|---|---|---|---|
| | | | | Beginning of the experiment | End of experiment | |
| 126 | Control | - | | s | s | |
| 142 | Control | - | | j | j | **dead** |
| 166 | Control | - | | s | s | |
| 124 | Exp. 1 | 1 h | | j | j | |
| 144 | Exp. 1 | 1 h | | j | j | |
| 167 | Exp. 1 | 1 h | yes | s | s | |
| 130 | Exp. 1 | 6 h | yes | s | s | |
| 137 | Exp. 1 | 6 h | | s | s | |
| 157 | Exp. 1 | 6 h | | j | j | |
| 127 | Exp. 1 | 12 h | | s | s | |
| 131 | Exp. 1 | 12 h | | s | s | |
| 155 | Exp. 1 | 12 h | | s | s | |
| 123 | Exp. 1 | 18 h | | s | s | |
| 145 | Exp. 1 | 18 h | | j | j | |
| 158 | Exp. 1 | 18 h | yes | j | j | |
| 122 | Exp. 2 | 1 h | | s | s | |
| 146 | Exp. 2 | 1 h | | j | j | |
| **163** | Exp. 2 | 1 h | yes | s | s | |
| 128 | Exp. 2 | 6 h | yes | s | s | |
| 134 | Exp. 2 | 6 h | | s | s | |
| 168 | Exp. 2 | 6 h | yes | j | j | |
| 125 | Exp. 2 | 12 h | | s | s | |
| 129 | Exp. 2 | 12 h | yes | j | j | **dead** |
| 154 | Exp. 2 | 12 h | | j | j | |
| 133 | Exp. 2 | 18 h | | s | s | |
| 152 | Exp. 2 | 18 h | yes | j | j | |
| 164 | Exp. 2 | 18 h | | j | j | |
| 132 | Exp. 3 | 8 h | | j | j | **dead** |
| 153 | Exp. 3 | 8 h | | j | j | After 3h: no food particle visible |
| 165 | Exp. 3 | 8 h | | j | j | After 3h: food particle still attached to the foram |
