# Supplementary material for: Ammonium is the preferred source of nitrogen for planktonic foraminifer and their dinoflagellate symbionts
Source: Proc Biol Sci. 2020 Jun 17;287(1929):20200620. doi: 10.1098/rspb.2020.0620 (PMC7329048; doi:10.1098/rspb.2020.0620)
Supplement: Table S2 [file rspb20200620supp7.pdf]

Table S2: Results of the t-tests used for the comparisons of the different cell compartments $\delta^{13}C$ between Experiment 1 and Experiment 2 determined for each time point.

Time points	Cell compartment	F test		T-test
		p-value	var.equal	p-value
1 h	dinoflagellates	0.005136	False	2.417e-07
	cytoplasm	2.877e-05	False	0.1271
	electron-opaque bodies	0.7653	True	0.4926
	lipid droplets	2.2e-16	False	0.0001699
6 h	dinoflagellates	0.9889	True	0.1623
	cytoplasm	0.4712	True	0.7762
	electron-opaque bodies	3.191e-06	False	0.8119
	lipid droplets	NA	NA	NA
12 h	dinoflagellates	NA	NA	NA
	cytoplasm	NA	NA	NA
	electron-opaque bodies	NA	NA	NA
	lipid droplets	NA	NA	NA
18 h	dinoflagellates	0.2899	True	0.05687
	cytoplasm	3.254e-06	False	0.8894
	electron-opaque bodies	0.0005748	False	0.06133
	lipid droplets	NA	NA	NA
