# Supplementary material for: Ammonium is the preferred source of nitrogen for planktonic foraminifer and their dinoflagellate symbionts
Source: Proc Biol Sci. 2020 Jun 17;287(1929):20200620. doi: 10.1098/rspb.2020.0620 (PMC7329048; doi:10.1098/rspb.2020.0620)
Supplement: Text S1 [file rspb20200620supp8.pdf]

Ammonium is the preferred source of nitrogen for planktonic foraminifer and their dinoflagellate symbionts

Charlotte LeKieffre, Howard J. Spero, Jennifer S. Fehrenbacher, Ann D. Russell, Haojia Ren, Emmanuelle Geslin, Anders Meibom

Text S1: Detailed protocol of the ^{15}N- and ^{13}C-labeled *Artemia salina* used in Exp. 3 were prepared according to the protocol described by Krueger et al. (2018).

Tetraselmis sp. microalgae were grown for 3 weeks in f/2-medium spiked with 0.882 mM $Na^{15}N0_3$ (99 % ^{15}N; Sigma-Aldrich) and 2 mM $NaH^{13}CO_3$ (99 % ^{13}C; Sigma Aldrich). Eggs of *Artemia salina* brine shrimp were hatched in seawater in outdoor aquarium tanks that were also supplemented with 2 mM $NaH^{13}CO_3$ and 3 µM $Na^{15}NO_3$ to maintain the ^{13}C- and ^{15}N-enrichment in the *Tetraselmis* algae. The *Artemia* nauplii were fed daily for nine days with the ^{15}N- and ^{13}C-enriched *Tetraselmis* algae, starting the second day after hatching. After a week, the *A. salina* were collected and freeze-dried. Some *A. salina* were subsampled at the end of the nine days and embedded for NanoSIMS analysis (see Krueger et al., 2018). This *Artemia* subsample yielded mean ^{13}C and ^{15}N enrichments that were 4.8 atom % and 33.8 atom %, respectively.

Reference:

Krueger, T., Bodin, J., Horwitz, N., Loussert-Fonta, C., Sakr, A., Escrig, S., Fine, M., Meibom, A., 2018. Temperature and feeding induce tissue level changes in autotrophic and heterotrophic nutrient allocation in the coral symbiosis – A NanoSIMS study. Scientific Reports 8, 12710. https://doi.org/10.1038/s41598-018-31094-1
