# Supplementary material for: Ammonium is the preferred source of nitrogen for planktonic foraminifer and their dinoflagellate symbionts
Source: Proc Biol Sci. 2020 Jun 17;287(1929):20200620. doi: 10.1098/rspb.2020.0620 (PMC7329048; doi:10.1098/rspb.2020.0620)
Supplement: Text S2 [file rspb20200620supp9.pdf]

Text S2: Detailed protocol of the chemical fixation, sample preparation for transmission electron microscopy and NanoSIMS imaging and analysis

At each sampling time point, selected *O. universa* specimens were individually transferred into 0.5 mL micro-centrifuge tubes containing filtered seawater with 4 % glutaraldehyde and 2 % paraformaldehyde (pH = 8.1), and fixed for 24 h at room temperature. After three successive seawater rinses, specimens were post-fixed with a solution of 2 % OsO_4 prepared in distilled water for 1 h, rinsed with deionized water and embedded in 3% Agar (Sigma-Aldrich, type VII-A, low gelling temperature) to preserve the location of the symbiotic dinoflagellates outside the foraminiferal shell. After decalcification in two successive 0.1 M EDTA baths (1 h and 48 h), samples were transferred through an ethanol dehydration series from 50 to 100 % EtOH. Finally, samples were embedded in LR white resin and polymerized in an oven at 70°C for 12h. Ultra-thin (70 nm) sections were collected from each specimen using an ultramicrotome (Reichert Ultracut S) equipped with a diamond knife (Diatome Ultra, 45). Ultra-thin sections were placed on formvar-carbon coated copper grids (Electron Microscopy Science, reference FCF200F1-Cu), stained with 2 % uranyl acetate for 10 min and observed with a transmission electron microscope (TEM, Philips 301 CM100, 80 kV). All preparation and imaging were conducted at the Electron Microscopy Facility of the University of Lausanne (Switzerland). Areas of interest for subsequent NanoSIMS imaging were selected from TEM images.

The sections used for TEM imaging were first coated with ca. 10 nm of gold, before analysis with a Cameca NanoSIMS 50L ion microprobe. Sections of *O. universa* were imaged by bombarding them with a Cs^- ion beam focused to a spot size of ca. 100 – 150 nm. The secondary molecular ions $^{12}C_2^-$, $^{13}C^{12}C^-$, $^{12}C^{14}N^-$ and $^{12}C^{15}N^-$ were collected simultaneously in electron multiplier detectors at a mass-resolution superior to 8000 (Cameca definition), enough to resolve potential interferences in the mass spectrum. Isotopic images were taken with a 256x256 pixel resolution and a size ranging from 10x10 to 45x45 µm. Quantified $^{13}C/^{12}C$ and $^{15}N/^{14}N$ ratios were obtained as follows:

$$\delta^{13}C\ (‰) = \left(\left({}^{13/12}C_{meas}/{}_{13/12}C_{control}\right) - 1\right) \times 10^3$$

$$\delta^{15}N(‰) = \left(\left({}^{15/14}N_{meas}/{}_{15/14}N_{control}\right) - 1\right) \times 10^3$$

where $^{13/12}C_{meas}$ and $^{15/14}N_{meas}$ are the $^{12}C^{13}C^-/^{12}C_2^-$ and $^{12}C^{15}N/^{12}C^{14}N$ ratios measured in the isotopically labeled samples, and $C_{control}$ and $N_{control}$ are the $^{12}C^{13}C^-/^{12}C_2^-$ and $^{12}C^{15}N/^{12}C^{14}N$ ratios measured in control samples from unspiked filtered seawater (see above), prepared and handled in an identical manner. The average ratios of the foraminifer endoplasm of three control specimens were used as standard ratios against which the experimental ^{13}C and ^{15}N-enrichments were quantified as shown in the equations above (i.e. not relative to the VPDB or atmospheric N_2 isotope references, respectively). It is noteworthy that chemical fixation and embedding processes are known to affect the isotopic content of cells, especially the ^{13}C-content, due to loss of soluble compounds and ^{12}C-dominated resin infiltration (Musat et al., 2014; Nomaki et al., 2018). However, as all the samples were prepared for TEM and NanoSIMS imaging following the same procedure, relative comparisons can be made.

NanoSIMS image processing was carried out using the software Look@NanoSIMS (Polerecky et al., 2012). ROIs were drawn on TEM images previously aligned with NanoSIMS images. Isotopic enrichments were calculated for the foraminifer cytoplasm, dinoflagellate, and cellular compartments. For the average isotopic enrichment of the foraminiferal cytoplasm, three circles of ca. 2 μm in diameter were drawn per image, avoiding highly enriched ^{13}C- and ^{15}N- organelles. Note that in contrast to what was done by LeKieffre et al., 2018b, the dinoflagellate ROIs were drawn around the entire dinoflagellate cell rather than around the starch grains in the symbiont cytosol. Thus, the values expressed here for the dinoflagellate symbionts are averages of the entire exposed surface of the microalgal cell. For each condition, time point, and type of cellular compartments, the average ^{13}C- and ^{15}N-enrichments were calculated based on 3 replicates (unless otherwise specified).

<u>References</u>:

LeKieffre, C., Spero, H.J., Russell, A.D., Fehrenbacher, J.S., Geslin, E., Meibom, A., 2018. Assimilation, translocation, and utilization of carbon between photosynthetic symbiotic dinoflagellates and their planktic foraminifera host. Marine Biology 165. https://doi.org/10.1007/s00227-018-3362-7

Musat, N., Stryhanyuk, H., Bombach, P., Adrian, L., Audinot, J.-N., Richnow, H.H., 2014. The effect of FISH and CARD-FISH on the isotopic composition of ^{13}C- and ^{15}N-labeled *Pseudomonas putida* cells measured by nanoSIMS. Systematic and Applied Microbiology 37, 267–276. https://doi.org/10.1016/j.syapm.2014.02.002

Nomaki, H., LeKieffre, C., Escrig, S., Meibom, A., Yagyu, S., Richardson, E.A., Matsuzaki, T., Murayama, M., Geslin, E., Bernhard, J.M., 2018. Innovative TEM-coupled approaches to study foraminiferal cells. Marine Micropaleontology 138, 90–104. https://doi.org/10.1016/j.marmicro.2017.10.002

Polerecky, L., Adam, B., Milucka, J., Musat, N., Vagner, T., Kuypers, M.M.M., 2012. Look@NanoSIMS – a tool for the analysis of nanoSIMS data in environmental microbiology. Environmental Microbiology 14, 1009–1023. https://doi.org/10.1111/j.1462-2920.2011.02681.x
