# Supplementary material for: Ammonium is the preferred source of nitrogen for planktonic foraminifer and their dinoflagellate symbionts
Source: Proc Biol Sci. 2020 Jun 17;287(1929):20200620. doi: 10.1098/rspb.2020.0620 (PMC7329048; doi:10.1098/rspb.2020.0620)
Supplement: Text S3 [file rspb20200620supp10.pdf]

Text S3: Statistical analysis

The $\delta^{13}C$ and $\delta^{15}N$ values of the dinoflagellate symbionts, foraminiferal cytoplasm, lipid droplets and electron-opaque bodies were obtained by calculating the average of ROIs (one ROI corresponding to one organelle or cell compartment) within each specimen, and then calculating the average of the three specimens for each time point. Thus, the errors bars shown are standard deviations representing the inter-specimen variability (n = 3 specimens, unless otherwise specified). However, statistical analysis was carried out on the total set of ROIs for each time point using a linear mixed-effect model taking into account pseudo-replication effects, followed by a Tukey multiple comparison test. The results of the linear mixed-effect model are listed in the supplementary file Text S4. Comparisons of $\delta^{13}C$ of the different cell compartments between Exp. 1 and Exp. 2 were performed with t tests for each cell compartment and each time point. The results of the t tests are listed in the Supplementary Table S2. All statistical analysis were performed with the RStudio software (RStudio Team, 2016) with the significance level set to $\alpha = 0.05$ (i.e., *p value* < 0.05).

No statistical comparisons were made for (1) the fibrillar body $\delta^{13}C$ and $\delta^{15}N$ enrichments as they were not analyzed in all the specimens and only a limited amount of data is available, (2) for the Exp. 3 feeding images as one specimen died during the experiment (see Supplementary Table S1) and only two replicates were available which is insufficient for robust statistical analysis, and (3) for $\delta^{13}C$ enrichments between the Exp. 1 and the Exp. 2 at t = 12 h were available for the Exp. 2 at this time point, and for the lipid droplets at t = 6, 12 and 18 h, as they could only be observed and analyzed in two replicates.
