# Supplementary material for: Ammonium is the preferred source of nitrogen for planktonic foraminifer and their dinoflagellate symbionts
Source: Proc Biol Sci. 2020 Jun 17;287(1929):20200620. doi: 10.1098/rspb.2020.0620 (PMC7329048; doi:10.1098/rspb.2020.0620)
Supplement: Text S4 [file rspb20200620supp11.pdf]

Text S4: Results of the different post-hoc Tukey multiple comparisons tests following the linear mixed-effects model analyses.

Experiment 1

- **$\delta^{13}C$ dinoflagellates**

```
    Simultaneous Tests for General Linear Hypotheses

Multiple Comparisons of Means: Tukey Contrasts

Fit: lme.formula(fixed = C ~ time, data = mydata, random = ~1 | Plotb)

Linear Hypotheses:
             Estimate Std. Error z value Pr(>|z|)
6 - 1 == 0    6053.23     658.48   9.193   <0.001 ***
12 - 1 == 0   6004.59     633.57   9.477   <0.001 ***
18 - 1 == 0   2466.11     655.43   3.763   <0.001 ***
12 - 6 == 0    -48.64     620.59  -0.078        1
18 - 6 == 0  -3587.13     642.89  -5.580   <0.001 ***
18 - 12 == 0 -3538.49     617.35  -5.732   <0.001 ***
---
Signif. codes:  0 ‘***’ 0.001 ‘**’ 0.01 ‘*’ 0.05 ‘.’ 0.1 ‘ ’ 1
(Adjusted p values reported -- single-step method)
```

- **$\delta^{13}C$ cytoplasm**

```
    Simultaneous Tests for General Linear Hypotheses

Multiple Comparisons of Means: Tukey Contrasts

Fit: lme.formula(fixed = C ~ time, data = mydata, random = ~1 | Plotb)

Linear Hypotheses:
             Estimate Std. Error z value Pr(>|z|)
6 - 1 == 0      140.4      193.7    0.73   0.8867
12 - 1 == 0     558.9      200.7    2.78   0.0271 *
18 - 1 == 0     638.5      196.0    3.26   0.0061 **
12 - 6 == 0     418.4      179.0    2.34   0.0894 .
18 - 6 == 0     498.0      173.8    2.87   0.0215 *
18 - 12 == 0     79.6      181.5    0.44   0.9717
---
Signif. codes:  0 ‘***’ 0.001 ‘**’ 0.01 ‘*’ 0.05 ‘.’ 0.1 ‘ ’ 1
(Adjusted p values reported -- single-step method)
```

- **$\delta^{13}C$ electron-opaque bodies**

```
    Simultaneous Tests for General Linear Hypotheses

Multiple Comparisons of Means: Tukey Contrasts

Fit: lme.formula(fixed = C ~ time, data = mydata, random = ~1 | Plotb)

Linear Hypotheses:
             Estimate Std. Error z value Pr(>|z|)
 6 - 1 == 0    723.520    107.369   6.739   <1e-05 ***
 12 - 1 == 0   784.552    110.900   7.074   <1e-05 ***
 18 - 1 == 0   794.398    125.749   6.317   <1e-05 ***
 12 - 6 == 0    61.032    103.948   0.587    0.936
 18 - 6 == 0    70.879    119.663   0.592    0.934
 18 - 12 == 0    9.847    122.840   0.080    1.000
---
Signif. codes:  0 '***' 0.001 '**' 0.01 '*' 0.05 '.' 0.1 ' ' 1
(Adjusted p values reported -- single-step method)
```

- **$\delta^{13}C$ lipid droplets**

```
    Simultaneous Tests for General Linear Hypotheses

Multiple Comparisons of Means: Tukey Contrasts

Fit: lme.formula(fixed = C ~ time, data = mydata, random = ~1 | Plotb)

Linear Hypotheses:
             Estimate Std. Error z value Pr(>|z|)
6 - 1 == 0        705        459    1.54   0.4151
12 - 1 == 0      2203        415    5.31   <0.001 ***
18 - 1 == 0       989        409    2.42   0.0727 .
12 - 6 == 0      1498        466    3.22   0.0073 **
18 - 6 == 0       284        460    0.62   0.9262
18 - 12 == 0    -1213        416   -2.91   0.0185 *
---
Signif. codes:  0 '***' 0.001 '**' 0.01 '*' 0.05 '.' 0.1 ' ' 1
(Adjusted p values reported -- single-step method)
```

- **$\delta^{15}N$ dinoflagellates**

```
    Simultaneous Tests for General Linear Hypotheses

Multiple Comparisons of Means: Tukey Contrasts

Fit: lme.formula(fixed = N ~ time, data = mydata, random = ~1 | Plotb)

Linear Hypotheses:
             Estimate Std. Error z value Pr(>|z|)
 6 - 1 == 0      10281       5060   2.032   0.1764
 12 - 1 == 0     12344       5010   2.464   0.0654 .
 18 - 1 == 0     20953       5053   4.147   <0.001 ***
 12 - 6 == 0      2063       4976   0.415   0.9760
 18 - 6 == 0     10672       5019   2.127   0.1447
 18 - 12 == 0     8609       4968   1.733   0.3065
---
Signif. codes:  0 '***' 0.001 '**' 0.01 '*' 0.05 '.' 0.1 ' ' 1
(Adjusted p values reported -- single-step method)
```

- **$\delta^{15}N$ cytoplasm**

```
    Simultaneous Tests for General Linear Hypotheses

Multiple Comparisons of Means: Tukey Contrasts

Fit: lme.formula(fixed = N ~ time, data = mydata, random = ~1 | Plotb)

Linear Hypotheses:
             Estimate Std. Error z value Pr(>|z|)
6 - 1 == 0       2355       1538    1.53   0.4169
12 - 1 == 0      4852       1608    3.02   0.0131 *
18 - 1 == 0      7068       1555    4.54   <0.001 ***
12 - 6 == 0      2497       1377    1.81   0.2656
18 - 6 == 0      4712       1315    3.58   0.0018 **
18 - 12 == 0     2216       1396    1.59   0.3845
---
Signif. codes:  0 '***' 0.001 '**' 0.01 '*' 0.05 '.' 0.1 ' ' 1
(Adjusted p values reported -- single-step method)
```

- **$\delta^{15}N$ electron-opaque bodies**

```
    Simultaneous Tests for General Linear Hypotheses

Multiple Comparisons of Means: Tukey Contrasts


Fit: lme.formula(fixed = N ~ time, data = mydata, random = ~1 | Plotb)

Linear Hypotheses:
             Estimate Std. Error z value Pr(>|z|)
 6 - 1 == 0       2789       1404   1.987   0.1929
 12 - 1 == 0      4077       1406   2.901   0.0193 *
 18 - 1 == 0      5650       1418   3.983   <0.001 ***
 12 - 6 == 0      1288       1402   0.919   0.7950
 18 - 6 == 0      2861       1415   2.021   0.1799
 18 - 12 == 0     1572       1417   1.110   0.6835
---
Signif. codes:  0 '***' 0.001 '**' 0.01 '*' 0.05 '.' 0.1 ' ' 1
(Adjusted p values reported -- single-step method)
```

Experiment 2

- **$\delta^{13}C$ dinoflagellates**

```
          Simultaneous Tests for General Linear Hypotheses

Multiple Comparisons of Means: Tukey Contrasts

Fit: lme.formula(fixed = C ~ time, data = mydata, random = ~1 | Plotb)

Linear Hypotheses:
             Estimate Std. Error z value Pr(>|z|)
6 - 1 == 0       5735        640    8.97   <0.001 ***
12 - 1 == 0      5545        705    7.87   <0.001 ***
18 - 1 == 0      1792        660    2.71    0.033 *
12 - 6 == 0      -190        669   -0.28    0.992
18 - 6 == 0     -3943        622   -6.34   <0.001 ***
18 - 12 == 0    -3753        688   -5.45   <0.001 ***
---
Signif. codes:  0 '***' 0.001 '**' 0.01 '*' 0.05 '.' 0.1 ' ' 1
(Adjusted p values reported -- single-step method)
```

- **$\delta^{13}C$ cytoplasm**

```
          Simultaneous Tests for General Linear Hypotheses

Multiple Comparisons of Means: Tukey Contrasts

Fit: lme.formula(fixed = C ~ time, data = mydata, random = ~1 | Plotb)

Linear Hypotheses:
             Estimate Std. Error z value Pr(>|z|)
6 - 1 == 0        184        105    1.74    0.302
12 - 1 == 0       525        120    4.38   <0.001 ***
18 - 1 == 0       773        106    7.31   <0.001 ***
12 - 6 == 0       341        119    2.87    0.021 *
18 - 6 == 0       590        105    5.64   <0.001 ***
18 - 12 == 0      249        119    2.09    0.156
---
Signif. codes:  0 '***' 0.001 '**' 0.01 '*' 0.05 '.' 0.1 ' ' 1
(Adjusted p values reported -- single-step method)
```

- **$\delta^{13}C$ electron-opaque bodies**

```
    Simultaneous Tests for General Linear Hypotheses

Multiple Comparisons of Means: Tukey Contrasts

Fit: lme.formula(fixed = C ~ time, data = mydata, random = ~1 | Plotb)

Linear Hypotheses:
             Estimate Std. Error z value Pr(>|z|)
  6 - 1 == 0      507.3      358.0   1.417   0.4868
  12 - 1 == 0    1055.0      404.3   2.609   0.0445 *
  18 - 1 == 0     924.8      358.0   2.583   0.0475 *
  12 - 6 == 0     547.7      368.1   1.488   0.4430
  18 - 6 == 0     417.5      316.6   1.319   0.5491
  18 - 12 == 0   -130.1      368.1  -0.354   0.9847
---
Signif. codes:  0 '***' 0.001 '**' 0.01 '*' 0.05 '.' 0.1 ' ' 1
(Adjusted p values reported -- single-step method)
```

- **$\delta^{13}C$ lipid droplets**

```
          Simultaneous Tests for General Linear Hypotheses

Multiple Comparisons of Means: Tukey Contrasts

Fit: lme.formula(fixed = C ~ time, data = mydata, random = ~1 | Plotb)

Linear Hypotheses:
             Estimate Std. Error z value Pr(>|z|)
6 - 1 == 0     1356.6       83.6   16.23   <1e-04 ***
12 - 1 == 0    1529.6      119.1   12.84   <1e-04 ***
18 - 1 == 0    1333.8       96.2   13.87   <1e-04 ***
12 - 6 == 0     173.1      125.9    1.37     0.51
18 - 6 == 0     -22.8      104.5   -0.22     1.00
18 - 12 == 0   -195.9      134.6   -1.45     0.46
---
Signif. codes:  0 '***' 0.001 '**' 0.01 '*' 0.05 '.' 0.1 ' ' 1
(Adjusted p values reported -- single-step method)
```

- **$\delta^{15}N$ dinoflagellates**

```
          Simultaneous Tests for General Linear Hypotheses

Multiple Comparisons of Means: Tukey Contrasts

Fit: lme.formula(fixed = N ~ time, data = mydata, random = ~1 | Plotb)

Linear Hypotheses:
             Estimate Std. Error z value Pr(>|z|)
6 - 1 == 0      178.3      128.2    1.39   0.5047
12 - 1 == 0     202.5      142.7    1.42   0.4866
18 - 1 == 0     429.8      129.2    3.33   0.0049 **
12 - 6 == 0      24.2      141.2    0.17   0.9982
18 - 6 == 0     251.5      127.6    1.97   0.1980
18 - 12 == 0    227.3      142.1    1.60   0.3780
---
Signif. codes:  0 '***' 0.001 '**' 0.01 '*' 0.05 '.' 0.1 ' ' 1
(Adjusted p values reported -- single-step method)
```

- **$\delta^{15}N$ cytoplasm**

```
          Simultaneous Tests for General Linear Hypotheses

Multiple Comparisons of Means: Tukey Contrasts

Fit: lme.formula(fixed = N ~ time, data = mydata, random = ~1 | Plotb)

Linear Hypotheses:
             Estimate Std. Error z value Pr(>|z|)
6 - 1 == 0      54.97      47.66    1.15   0.6553
12 - 1 == 0    157.49      53.89    2.92   0.0179 *
18 - 1 == 0    149.45      47.77    3.13   0.0096 **
12 - 6 == 0    102.51      53.56    1.91   0.2212
18 - 6 == 0     94.48      47.41    1.99   0.1899
18 - 12 == 0    -8.03      53.66   -0.15   0.9988
---
Signif. codes:  0 '***' 0.001 '**' 0.01 '*' 0.05 '.' 0.1 ' ' 1
(Adjusted p values reported -- single-step method)
```

- **$\delta^{15}N$ electron-opaque bodies**

```
    Simultaneous Tests for General Linear Hypotheses

Multiple Comparisons of Means: Tukey Contrasts

Fit: lme.formula(fixed = N ~ time, data = mydata, random = ~1 | Plotb)

Linear Hypotheses:
             Estimate Std. Error z value Pr(>|z|)
 6 - 1 == 0      17.70      47.68   0.371 0.982100
 12 - 1 == 0    244.87      59.28   4.131 0.000205 ***
 18 - 1 == 0     41.45      48.97   0.847 0.828688
 12 - 6 == 0    227.16      49.80   4.561  < 1e-04 ***
 18 - 6 == 0     23.75      36.93   0.643 0.916138
 18 - 12 == 0  -203.42      51.03  -3.986 0.000328 ***
---
Signif. codes:  0 '***' 0.001 '**' 0.01 '*' 0.05 '.' 0.1 ' ' 1
(Adjusted p values reported -- single-step method)
```
